# Could the Microbiota Be a Predictive Factor for the Clinical Response to Probiotic Supplementation in IBS-D? A Cohort Study

**DOI:** 10.3390/microorganisms11020277

**Published:** 2023-01-20

**Authors:** Justine Marchix, Lucille Quénéhervé, Philippe Bordron, Philippe Aubert, Tony Durand, Thibauld Oullier, Claude Blondeau, Samira Ait Abdellah, Stanislas Bruley des Varannes, Samuel Chaffron, Emmanuel Coron, Michel Neunlist

**Affiliations:** 1 Nantes Université, CHU Nantes, INSERM, The Enteric Nervous System in Gut and Brain Diseases, IMAD, F-44000 Nantes, France; 2 Gastroenterology Department, University Hospital of Brest, 29200 Brest, France; 3 PiLeJe Laboratoire, 31-35 rue de la Fédération, 75015 Paris, France; 4 Nantes Université, CHU Nantes, INSERM, Département de Gastroentérologie, CIC 1413, IMAD, F-44000 Nantes, France; 5 Nantes Université, École Centrale Nantes, CNRS, LS2N, UMR 6004, F-44000 Nantes, France; 6 Gastroenterology and Hepatology Department, University Hospital of Geneva (HUG), 1211 Geneva, Switzerland

**Keywords:** irritable bowel syndrome, diarrhoea, intestinal permeability, probiotics, microbiota

## Abstract

Background: Increasing evidence suggests the beneficial effects of probiotics in irritable bowel syndrome (IBS), but little is known about how they can impact the gut microbiota. Our objective was to evaluate the effects of a multistrain probiotic on IBS symptoms, gut permeability and gut microbiota in patients with diarrhoea-predominant IBS (IBS-D). Methods: Adults with IBS-D were enrolled in an open-label trial to receive a multistrain probiotic for 4 weeks. Abdominal pain, stool frequency, quality of life, gut permeability, and the luminal and adherent microbiota from colonic biopsies were evaluated before and after supplementation. Results: Probiotics significantly improved symptoms and quality of life, despite having no impact on permeability in the global population. In the population stratified by the response, the diarrhoea responders displayed reduced colonic permeability after supplementation. The luminal and adherent microbiota were specifically altered depending on the patients’ clinical responses regarding pain and diarrhoea. Interestingly, we identified a microbial signature in IBS-D patients that could predict a response or lack of response to supplementation. Conclusions: The multistrain probiotic improved the symptoms of IBS-D patients and induced distinct effects on the gut microbiota according to the patient’s clinical response and initial microbiota composition. Our study further supports the need to develop individualised probiotic-based approaches regarding IBS.

## 1. Introduction

Irritable bowel syndrome (IBS) is one of the most common functional gastrointestinal disorders encountered by primary care physicians and gastroenterologists, with an estimated prevalence of 3–11%, depending on the geographical region and assessment criteria [1,2]. The pathogenesis of IBS is multifactorial and remains poorly defined. However, increasing evidence suggests that alterations of the gut microbiota may favour the development and persistence of IBS [3,4,5]. A meta-analysis showed a significantly lower abundance of *Lactobacillus*, *Bifidobacterium* and *Faecalibacterium prausnitzii* in IBS patients compared to healthy controls [6]. These changes in the microbiota composition could notably contribute to increased permeability of the intestinal epithelial barrier, as observed in some IBS patients [7,8,9,10].

These findings have encouraged the evaluation of probiotics in the management of IBS as they represent an attractive approach, given their multiple mechanisms of action. These include the production of antibacterial substances, the inhibition of pathogens and antagonism of toxins, anti-inflammatory properties, the beneficial effects on intestinal barrier function, and the inhibitory effects on visceral hypersensitivity [8,9,10,11]. Recent meta-analyses of randomised controlled trials indicated a significant beneficial effect of probiotics on IBS symptoms overall, including abdominal pain, with a rate of adverse events not significantly higher than those seen with the placebo [12,13,14,15,16]. In particular, several randomised placebo-controlled trials have shown a reduction in symptom severity and an improvement in quality of life after supplementation with the probiotic strains of *Lactobacillus* and *Bifidobacterium* species [1,11,17,18,19,20].

A major challenge in treating IBS is the diversity of symptoms and their underlying mechanisms, leading to a search for more personalised therapeutic approaches. In line with this aim, the American Gastroenterological Association (AGA) has just issued recommendations to differentiate pharmacological treatment for IBS according to its subtype, namely diarrhoea-predominant (IBS-D) or constipation-predominant (IBS-C) IBS [21]. Whether such personalised approaches could be proposed using probiotics remains to be fully demonstrated. Recent data suggest that specific microbiota signatures are associated with IBS subtypes. A meta-analysis by Liu et al. [6] revealed a clear decrease in the *Lactobacillus* and *Bifidobacterium* species in patients with IBS-D, whereas no significant difference was seen between the IBS-C patients and healthy control subjects. Tap et al. identified a microbiota signature associated with IBS severity [22]. However, the possibility of identifying the microbiota signatures predictive of treatment efficacy in IBS, in particular that of probiotics, remains largely unexplored.

In this context, the first objective of our study was to identify the clinical, functional, and microbial effects of a multistrain probiotic in patients suffering from IBS-D. The multistrain probiotic tested (Lactibiane Tolérance^®^, PiLeJe Laboratoire, Paris, France), comprising a mixture of five lactic acid bacteria, has already been shown to prevent epithelial barrier alteration in various in vitro and in vivo models of IBS [23]. Our second objective was to determine whether we could identify a microbial signature that could predict the clinical response to this probiotic supplementation in IBS-D patients.

## 2. Materials and Methods

### 2.1. Design and Ethics Statement

This was a single-arm, open-label study conducted in a single centre in France (the Hepato-Gastroenterology Clinical Investigation Centre of the University Hospital of Nantes, France). The study was approved by the local Ethics Committee on 22 January 2016 and was performed in accordance with the ethical standards laid down in the Declaration of Helsinki and the rules of Good Clinical Practice (GCP). It was registered on the ClinicalTrials.gov site on 5 April 2016 (registration number NCT02728063).

### 2.2. Supplementation

The tested multistrain probiotic, Lactibiane Tolérance^®^ (PiLeJe Laboratoire, France), comprises a mixture of five viable lyophilized lactic acid bacteria (*Bifidobacterium lactis* LA 303, *B. lactis* LA 304, *Lactobacillus acidophilus* LA 201, *L. plantarum* LA 301, and *L. salivarius* LA 302) at a total concentration of 10 × 10^9^ colony-forming units (CFU) per capsule, with corn starch and magnesium stearate as the excipients. Patients were to take two capsules per day, which were to be swallowed with a large glass of water in the morning on an empty stomach for 4 weeks. At the first study visit (V1), patients were provided with a sufficient supply of capsules for 30 days of supplementation.

### 2.3. Patients

To be eligible for enrolment, male or female patients had to (i) be between 18 and 75 years of age; (ii) present IBS-D according to the Rome III criteria; (iii) be in a general and mental state of health compatible with participation in the study; (iv) agree to maintain their usual lifestyle throughout the study; and (v) signify their willingness to participate in the study by signing and dating an informed consent form.

Patients were not included in the study if they had (i) a history of hypersensitivity to the test product or to fluorescein; (ii) an immunodeficiency or a serious or progressive illness; (iii) a metabolic disorder or an inflammatory bowel disease affecting bowel movements or nutrient absorption, such as diabetes, hyperthyroidism, coeliac disease or Crohn’s disease; (iv) an existing medical history or condition that, in the investigator’s opinion, was likely to interfere with the results of the study or expose the patient to additional risk; (v) a treatment that might interfere with the study. Pregnant or breastfeeding women were also ineligible for inclusion.

### 2.4. Study Procedure

The study comprised three visits, one for patient screening (V0; 2 to 6 weeks before V1), one for inclusion (V1) and one at the end of the 4-week supplementation phase ± 2 days (V2).

At V0, a medical examination was performed, socio-demographic data, medical and surgical history, and information on lifestyle and concomitant treatments were recorded, and the eligibility criteria were checked. If their eligibility was confirmed, the patients were enrolled and invited to return for V1 and V2 with the following recommendations: (i) not to change their lifestyle (especially their physical activity) and eating habits; (ii) to stop taking anti-diarrhoeal medication, but not to change their other treatments unless these were specifically prohibited in the study (antispasmodics were accepted); (iii) not to start any new treatment with psychotropic drugs, antihistamines, antibiotics or anti-inflammatory drugs (aspirin or ibuprofen) unless absolutely necessary; (iv) not to consume foods or food supplements enriched with prebiotic fibres, probiotics, omega fatty acids, or other substances, such as vitamins and minerals; (v) not to consume food products containing polyols, such as chocolate, sweets or “sugar-free” chewing gum, during the 48 h preceding V1 and V2; (vi) not to consume alcohol during the 48 h prior to V1 and V2. In addition, on arrival at V1 and V2, patients were to have fasted for at least 8 h.

At V1 and V2, the investigator checked that these recommendations had been followed since the previous visit, performed a medical examination and a urine pregnancy test for women of childbearing age, recorded concomitant treatments and adverse events, and verified that the patient still met the eligibility criteria. If he/she was still eligible, the following procedures and tests were performed: withdrawal of a blood sample of ≤30 mL; preparation for rectosigmoidoscopy using a rectal enema solution (if not administered at home before the visit); rectosigmoidoscopy with confocal endomicroscopy and fluorescein injection; colonic biopsies taken during this same rectosigmoidoscopy; and a 5-h dynamic lactulose/mannitol test. During these visits, the patients completed the Gastrointestinal Quality of Life Index (GIQLI [24]) comprising five subscales (Symptoms, Emotions, Physical function, Social function and Medical treatment) and a global score. In addition, for 7 days prior to each visit, the patients were to evaluate their worst abdominal pain and abdominal discomfort on a Likert scale (0–10) and their stool frequency and consistency, according to the Bristol Stool Scale. Compliance and adverse events were also recorded.

### 2.5. Definition of Clinical Response

The clinical response was defined on the basis of two criteria: abdominal pain intensity and stool frequency. A decrease of ≥30% in the mean weekly abdominal pain score between V1 and V2 was considered a response with regard to the pain criterion [25]. A decrease of ≥50% in the mean number of days per week on which the patient reported stools of type 6 or 7 on the Bristol Stool Scale was considered a response regarding the diarrhoea criterion [25]. Based on these definitions, patients were considered responders (R) or non-responders (NR) to the probiotic supplementation with regards to pain or diarrhoea criteria.

### 2.6. Evaluation of Intestinal Permeability

Intestinal permeability was assessed in vivo by the percentage urinary excretion of lactulose 2 to 4 h after oral intake of a lactulose-mannitol solution, reflecting a small intestine transit at V1 and V2. After oral intake of the lactulose and mannitol solution, urines were collected every hour for 5 h in separate jars [26]. The percentage of urinary excretion of lactulose from 2 to 4 h after ingestion of the lactulose-mannitol mixture was calculated by linear regression.

The ex vivo intestinal permeability was measured from three different biopsies per patient in Ussing chambers, as previously described [27]. Briefly, each biopsy was placed in an ice-cold Krebs solution and micro-dissected prior to being mounted in Ussing chambers. Tissues were bathed with F12-supplemented Dulbecco’s Modified Eagle medium (Thermo Fisher ScientificSaint-Aubin, France) containing 0.1% fetal bovine serum, 2 mM Glutamine and 45 g/L of NaHCO_3_. The medium was continuously oxygenated with a gas flow of 95% O_2_/5% CO_2_ and maintained at 37 °C. Fluorescein-5.6 sulfonic acid (FSA, 1 mg.mL^−1^, 400 Da Thermo Fisher Scientific, Saint-Aubin, France) and Horseradish Peroxidase (HRP, 3.75 mg.mL^−1^, 44 kDa, Sigma Aldrich, St. Quentin-Fallavier, France) were used to determine the intestinal paracellular and transcellular permeability, respectively. The flux of FSA was evaluated by the fluorescence level, measured on a basolateral chamber over 3 h using a spectrofluorometer (λex. 488 nm, λem. 520 nm) (Varioskan, Thermo Fisher Scientific, Saint-Aubin, France). The HRP activity was determined by an enzymatic assay based on tetramethylbenzidine (TMB) substrate (#555214, Becton-Dickinson), and the optical density was measured using a spectrophotometer microplate reader (λ 450 nm). The ex vivo paracellular and transcellular permeabilities were determined by averaging the SFA and HRP fluxes of the three biopsies.

### 2.7. Rectosigmoidoscopy and Probe-Based Confocal Laser Endomicroscopy (pCLE) Procedure

The rectosigmoidoscopy was performed by a single endoscopist in non-sedated patients after a distal colon cleansing with an enema using a standard colonoscope (EC530, Fujifilm, Japan). The colonic mucosa was examined up to 35 cm from the anal margin. Standard biopsies were then taken for a conventional routine histology and laboratory analysis. A pCLE recording was performed using a dedicated confocal laser endomicroscopy system comprising a portable laser station (Cellvizio; Mauna Kea Technologies, Paris, France) and an endoscopic probe (Coloflex; Mauna Kea Technologies). After an intravenous injection of 5 mL of a 10% fluorescein sodium solution, the probe was threaded through the operating channel of the endoscope and positioned on the colonic mucosa, after which the recording was started within 10 min, as previously reported [28]. The choice of the colonic areas subjected to the CLE imaging was left to the endoscopist’s discretion, as the mucosa had a normal aspect in all patients. A semi-automated and reproducible method of reading the confocal endomicroscopy films developed by our group was used [28].

### 2.8. Microbiota Analysis

#### 2.8.1. 16S rRNA Sequencing

Human colonic biopsies and stool samples were used to determine the luminal and adherent microbiota, respectively. Microbial genomic DNA extraction (using a Maxwell^®^ 16 instrument) and 16S metabarcoding sequencing were performed by Biofortis (Nantes, France). Both 16S rRNA libraries were constructed to target and amplify the V3–V4 regions of the 16S rRNA gene using the S-D-Bact-0341-b-S-17 and S-D-Bact-0785-a-A-21 adapters. The paired-end (2 × 250 cycle mode) sequencing was performed using the Illumina Miseq platform (MiSeq V2 reagent kit, Illumina Inc., San Diego, CA, USA).

#### 2.8.2. 16S Data Analysis

The luminal and adherent microbiota data analyses were conducted independently. The raw reads were processed using microSysMics (https://bio.tools/microSysMics, accessed on 27 September 2021), a workflow system based on the Quantitative Insights into Microbial Ecology 2 (Qiime2) toolbox [29]. Each ASV was assigned a taxonomic rank using the sklearn classifier from qiime2-2020.6 [29] with the SILVA 132 taxonomy database. Diversity metrics and composition analyses were performed on the rarefied ASV matrix. A sub-sampling depth of 48,049 sequences per sample was chosen for the luminal samples and a depth of 2500 sequences for the adherent samples for a final rarefied dataset of 52 samples (100% of the luminal samples) and 36 samples (78% of the adherent samples), respectively. The ASVs were filtered using a prevalence cut-off of 20%.

Three distinct analyses were conducted: an initial analysis to compare the overall samples from V1 to V2 and two stratified analyses according to the patient’s response with regard to abdominal pain and diarrhoea. The α-diversity was estimated according to three metrics: (a) richness (number of distinct ASVs observed); (b) evenness, based on the Pielou index; and (c) the Shannon index of diversity, using the R 4.0.3 package. The statistical analysis was performed using the Kruskal–Wallis test to compare the diversity between the groups. The β–diversity analysis was calculated according to the Bray–Curtis distance and visualised in PCoA by using the Vegan 2.5-7 package with R 4.0.3. The difference in microbial β-diversity was tested using the permutational multivariate analysis of variance (PERMANOVA), computed with the adonis function of the Vegan package with default parameters (999 permutations). Differential abundance analyses were performed at the genus and amplicon sequence variant (ASV) taxonomic levels using the DESeq2 1.26.0 post-count method [30]. Only the results with an adjusted *p*-value of <0.05 after the Benjamini–Hochberg correction (false discovery rate, FDR) were considered.

### 2.9. Statistical Analysis

The analysis of clinical data was performed on the intention-to-treat (ITT) and per-protocol (PP) populations using Prism 7.04. The categorical variables were expressed as numbers and proportions, and the quantitative variables as means (±standard deviation). Quantitative data were compared between V1 and V2 using a paired *t*-test or a non-parametric paired *t*-test (Wilcoxon test) if appropriate. The normal distribution of data was evaluated by the Agostino and Pearson test. A *p*-value less than 0.05 was considered significant.

## 3. Results

### 3.1. Patient Population

**Figure 1:**
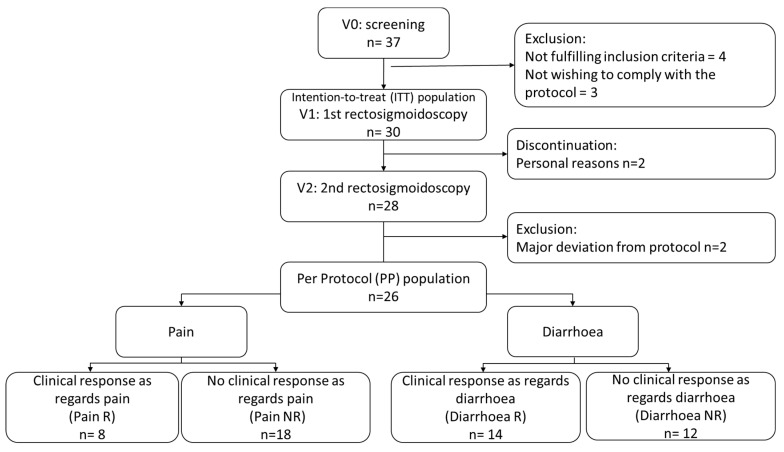
Study flow chart. ITT: intention-to-treat; PP: per-protocol; R: responders; NR: non-responders.

A total of 37 patients were assessed for eligibility (Figure 1), of whom 7 were excluded because they did not fulfil the inclusion criteria or did not wish to comply with the protocol procedures. Consequently, 30 patients (56.7% [n = 17] female; mean age 39.1 ± 14.5 years) were enrolled in the study and received at least one dose of the study product (ITT population). A total of 28 patients completed the study, with 2 patients discontinuing the study prematurely for personal reasons. Two patients presenting with major deviations from the protocol were excluded from the PP population (n = 26). The clinical results presented here are those obtained in the PP population, as the microbiota analyses were performed in this population. However, similar clinical results were obtained in the ITT population.

### 3.2. Clinical Response to a 4-Week Supplementation with the Multistrain Probiotic

**Figure 2:**
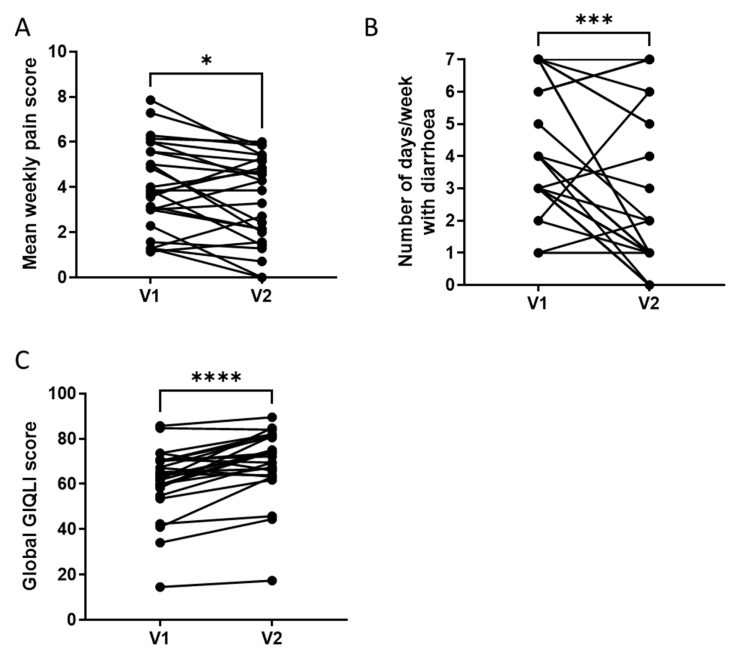
Clinical response to a 4-week supplementation with the multistrain probiotic in the per-protocol population. (**A**) Decrease in mean weekly abdominal pain score between V1 and V2. Paired *t*-test; *: *p* < 0.05; (**B**) decrease in mean number of days per week with diarrhoea (score of 6 or 7 on the Bristol Stool Scale) between V1 and V2; (**C**) increase in mean global Gastrointestinal Quality of Life Index (GIQLI) score between V1 and V2; Wilcoxon test, ***: *p* < 0.001; ****: *p* < 0.0001.

Between V1 and V2, the mean weekly abdominal pain score decreased significantly (from 4.1 ± 1.9 at V1 to 3.5 ± 1.9 at V2, *p* = 0.023; Figure 2A), as did the mean weekly intensity of abdominal discomfort (from 4.5 ± 1.9 at V1 to 3.8 ± 2.0 at V2, *p* = 0.01), and the mean number of days per week with diarrhoea (from 4.2 ± 1.6 at V1 to 2.5 ± 2.4 at V2, *p* = 0.0008; Figure 2B).

Among the 26 patients comprising the PP population, 8 presented a ≥30% decrease in their weekly abdominal pain score and were identified as Pain Responders [Pain R] and 14 achieved a ≥50% reduction in the number of days with diarrhoea and were identified as Diarrhoea Responders [Diarrhoea R].

Patient quality of life improved during the study, with the global GIQLI score increasing significantly between V1 and V2 from 60.6 ± 14.4 to 68.7 ± 14.95 (*p* < 0.0001; Figure 2C). Furthermore, the mean GIQLI score for the Symptom, Emotion, Physical function and Social function sub-dimensions increased significantly between V1 and V2, whereas there was no significant difference in the mean GIQLI score for the Medical treatment dimension, signifying that the patients were not inconvenienced by the supplement (Table 1).

### 3.3. Effects of a 4-Week Supplementation with the Multistrain Probiotic on Intestinal Permeability

**Figure 3:**
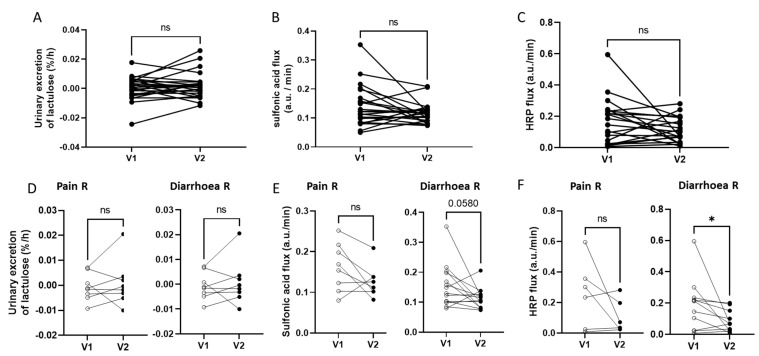
Impact of a 4-week supplementation with the multistrain probiotic on intestinal permeability in the per-protocol population as a whole (**A**–**C**) and in responders in terms of Pain (Pain R) and Diarrhoea (Diarrhoea R) (**D**–**F**). (**A**,**D**) In vivo permeability, measured by the lactulose/mannitol test; the slope of urinary excretion of lactulose from 2 to 4 h after ingestion between V1 and V2; (**B**,**E**) ex vivo paracellular permeability, assessed by the acid flux in colonic biopsies at V1 and V2; (**C**,**F**) ex vivo transcellular permeability, measured by the horseradish peroxidase (HRP) flux in colonic biopsies at V1 and V2; ns: not significant. Wilcoxon test, *: *p* < 0.05.

The in vivo permeability, estimated by the urinary excretion of lactulose from 2 to 4 h after ingestion, remained unchanged between V1 (0.001 ± 0.0076%/h) and V2 (0.002 ± 0.0085%/h, *p* = 0.402) (Figure 3A). Furthermore, neither the ex vivo colonic paracellular permeability, assessed by the sulfonic acid flux (V1: 0.16 ± 0.13 a.u./min; V2: 0.2 ± 0.44 a.u./min; *p* = 0.502; Figure 3B), nor transcellular permeability, estimated by the HRP flux (V1: 0.17 ± 0.14 ng/mL/min; V2: 0.11 ± 0.08 ng/mL/min; *p* = 0.066; Figure 3C), was modified after supplementation.

The analysis of colonic permeability, according to the clinical responses of patients, revealed differences between the pain responders (Pain R) and diarrhoea responders (Diarrhoea R) (Figure 3D–F). The paracellular and transcellular permeability remained unchanged in Pain R following supplementation (Figure 3E,F). In contrast, in Diarrhoea R, the paracellular permeability tended to decrease between V1 and V2 (0.16 ± 0.07 a.u./min at V1 versus 0.11 ± 0.03 at V2; *p* = 0.058, Figure 3E) and the transcellular permeability was significantly reduced after supplementation (0.18 ± 0.15 ng/mL/min at V1 versus 0.09 ± 0.07 ng/mL/min at V2; *p* = 0.024, Figure 3F). The in vivo permeability did not differ between V1 and V2 in either of the responder subgroups (Figure 3D).

### 3.4. Effects of a 4-Week Supplementation with the Multistrain Probiotic on pCLE Parameters

No significant difference between V1 and V2 was seen in terms of the architectural parameters, crypt distribution and vascular parameters overall (Table S1). No difference in the mucosal morphological parameters was observed in either Pain R or Diarrhoea R.

### 3.5. Effects of a 4-Week Supplementation with the Multistrain Probiotic on the Adherent and Luminal Gut Microbiota

**Figure 4:**
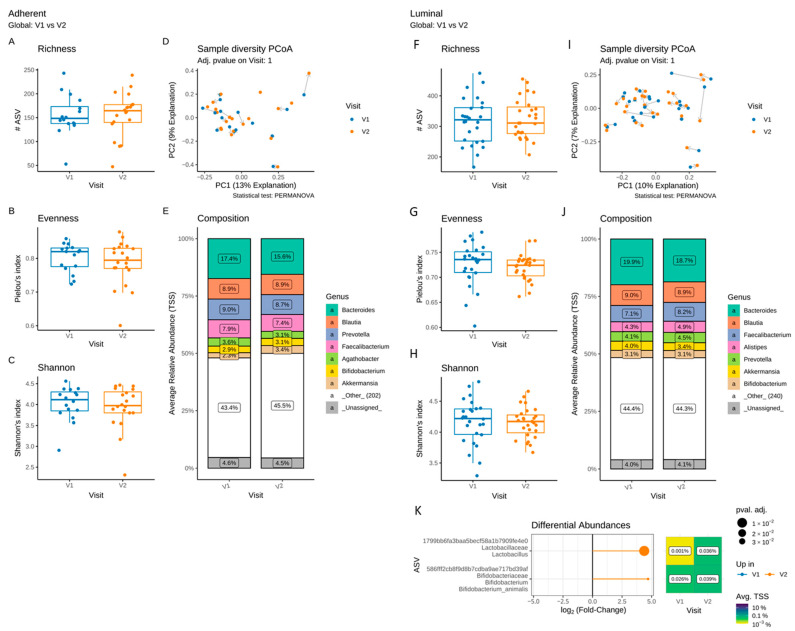
Impact of a 4-week supplementation with the multistrain probiotic on the adherent (**A**–**E**) and luminal (**F**–**K**) gut microbiota. (**A**–**C**,**F**–**H**) α-diversity, estimated by the richness (number of observed ASVs) (**A**,**F**), evenness (Pielou’s index) (**B**,**G**) and Shannon’s index (**C**,**H**) at V1 and V2; (**D**,**I**) principal coordinate analysis (PCoA) of bacterial β-diversity, measured according to Bray–Curtis distance; (**E**,**J**) bacterial taxonomic profile at genus level before (V1) and after (V2) supplementation; (**K**) differential abundance analysis of the luminal microbiota at the ASV level at V1 and V2. Only significant data are presented as Log2 of the fold of change (FC) according to DESeq2 analysis. *Transcription start sites (TSS)*.

We compared the evolution of the luminal and adherent microbiota composition between V1 and V2 in the PP population (Figure 4).

In the adherent (Figure 4A–C) and luminal (Figure 4F–I) microbiota, the α-diversity, estimated by the richness, evenness and Shannon indices, remained unchanged after supplementation, as did the β-diversity (Figure 4D). No significant difference in the composition of the adherent microbiota was detected between V1 and V2 (Figure 4E). No differences in the proportion of taxa at the phylum to the genus level were observed in the adherent microbiota (Figure 4J). However, in the luminal microbiota, significant increases in the ASVs assigned to *Lactobacillus* spp. and *Bifidobacterium animalis* were identified following supplementation (Figure 4K) and corresponded to the strains of the multistrain probiotic.

### 3.6. Differential Luminal and Adherent Microbiota Composition in Pain R and Diarrhoea R

As gut permeability was differentially affected in Pain R and Diarrhoea R, we investigated whether the gut microbiota composition varied between these subgroups. Second, we questioned whether the microbiota composition before supplementation (V1) could predict the response in terms of abdominal pain and diarrhoea.

#### 3.6.1. Microbiota Changes in Pain R

**Figure 5:**
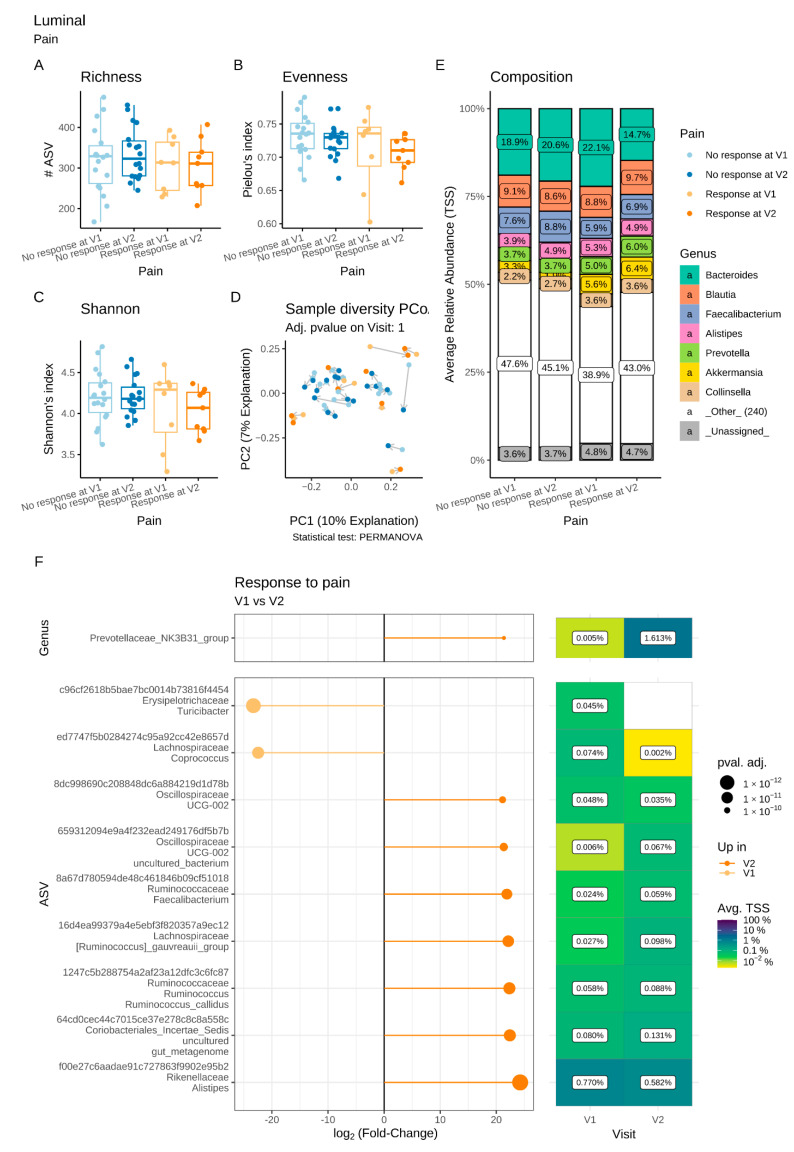
Impact of a 4-week supplementation with the multistrain probiotic on the luminal gut microbiota in Pain responders (R) and non-responders (NR). A-C: α-diversity estimated by the richness (number of observed ASVs) (**A**), evenness (Pielou’s index) (**B**) and Shannon’s index (**C**) at V1 and V2; (**D**) Principal coordinate analysis (PCoA) of bacterial β-diversity measured according to the Bray-Curtis distance; (**E**) Bacterial taxonomic profile at genus level prior (V1) and after supplementation (V2); (**F**) Differential abundance analysis at the genus and ASV levels at V1 and V2 in Pain R. Only significant data are presented as Log2 of the fold of change (FC) according to DESeq2 analysis. *Transcription start sites (TSS)*.

Regarding the luminal microbiota, the α- and β-diversity indices were similar in Pain R and Pain NR (non-responders) at V1 and V2 (Figure 5A–D). However, the differential abundance analysis revealed significant differences in the bacterial composition in Pain R between the two visits that were not observed in Pain NR (Figure 5E,F). At the genus level, a significant increase in *Prevotella* NK3B31 was observed between V1 and V2 (Figure 5F). In addition, significant increases in the ASVs assigned to *Oscillospiraceae* spp., *Coriobacteriales* spp., *Ruminococcaceae* genera (*Faecalibacterium* and *Ruminococcus*), *Alistipes* and *R. gauvreauii,* and a concomitant reduction of *Turibacter* and *Coprococcus* were observed in Pain R at V2 compared to V1.

**Figure 6:**
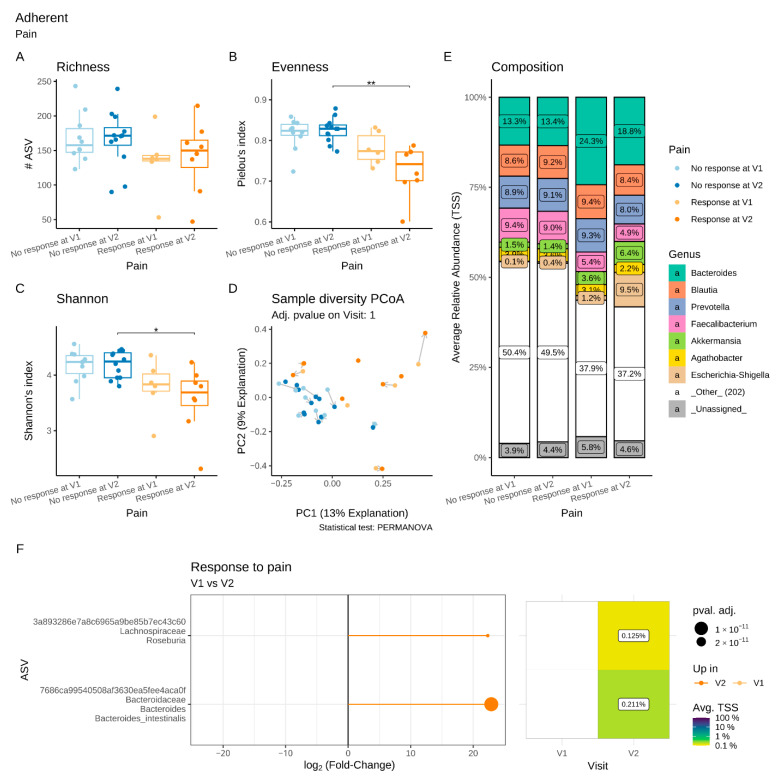
Impact of a 4-week supplementation with the multistrain probiotic on the adherent gut microbiota in Pain responders (R) and non-responders (NR). (**A**–**C**): α-diversity, estimated by the richness (number of observed ASVs) (**A**), evenness (Pielou’s index) (**B**) and Shannon’s index (**C**); (**D**) principal coordinate analysis (PCoA) of bacterial β-diversity, measured according to the Bray–Curtis distance; (**E**) bacterial taxonomic profile at genus level before (V1) and after (V2) supplementation; (**F**) differential abundance analysis at the ASV level at V1 and V2 in Pain R. Only significant data are presented as Log2 of the fold of change (FC) according to DESeq2 analysis. *: *p* < 0.05, **: *p* < 0.01 *Transcription start sites (TSS)*.

Regarding the adherent microbiota, there was a significant decrease in evenness and Shannon’s indices in Pain R compared to Pain NR at V2, not accompanied by changes in either richness or β-diversity (Figure 6A–D). Differential abundance analysis revealed a significant increase in two ASVs, assigned to the genus *Roseburia* and the species *Bacteroides intestinalis,* respectively, between V1 and V2 in Pain R, whereas no changes were observed in Pain NR (Figure 6E,F).

**Figure 7:**
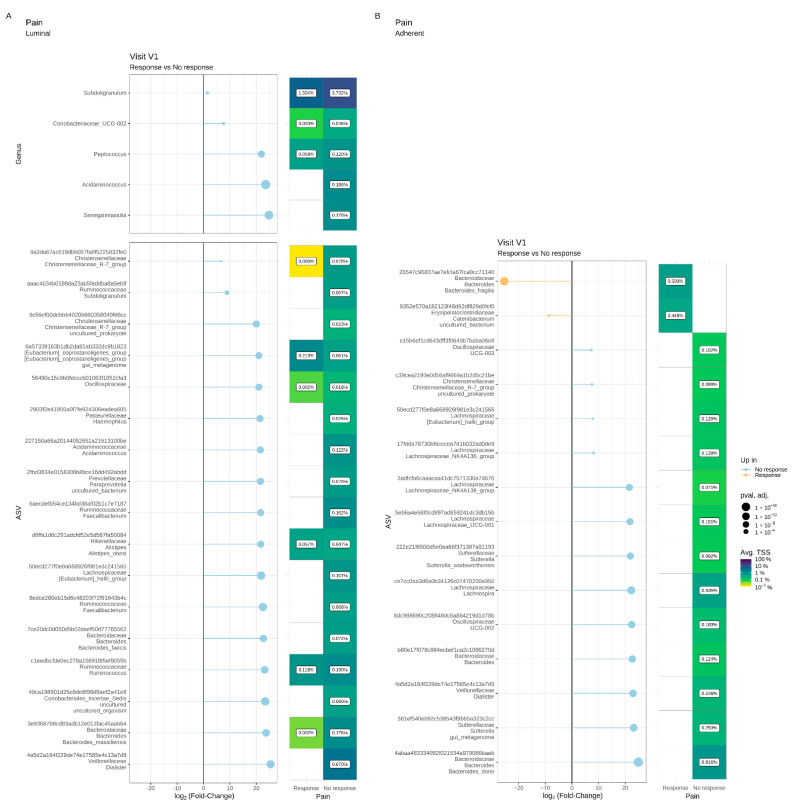
Differential abundance analysis of the luminal (**A**) and adherent (**B**) microbiota at the genus and ASV levels prior to supplementation (V1) in Pain responders and Pain non-responders. Only significant data are presented as Log2 of the fold of change (FC) according to DESeq2 analysis. *Transcription start sites (TSS)*.

A luminal microbiota signature that could be predictive of the absence to response in terms of abdominal pain was identified at V1 (Figure 7A). Compared to Pain R, Pain NR showed significant enrichment in the genera *Subdoligranulum*, *Coriobacteriaceae UCG-002*, *Peptococcus*, *Acidaminococcus* and *Senegalimassilia*. Similarly, 17 ASVs assigned to various families, including *Christensenellaceae*, *Ruminococcaceae* and *Pasteurellaceae*, were significantly increased in Pain NR compared to Pain R at V1. We also identified a putative adherent microbial signature at V1 that could predict pain response (Figure 7B). Thirteen ASVs assigned to various families (including *Christensenellaceae*, *Sutturellaceae*, *Bacteirodaceae* and *Lachnospiraceae*) were significantly enriched in Pain NR compared to Pain R at V1. In contrast, two ASVs belonging to *B. fragilis* and the *Catenibacterium* genus, respectively, were more abundant in Pain R when compared to Pain NR at V1.

#### 3.6.2. Microbiota Changes in Diarrhoea R

**Figure 8:**
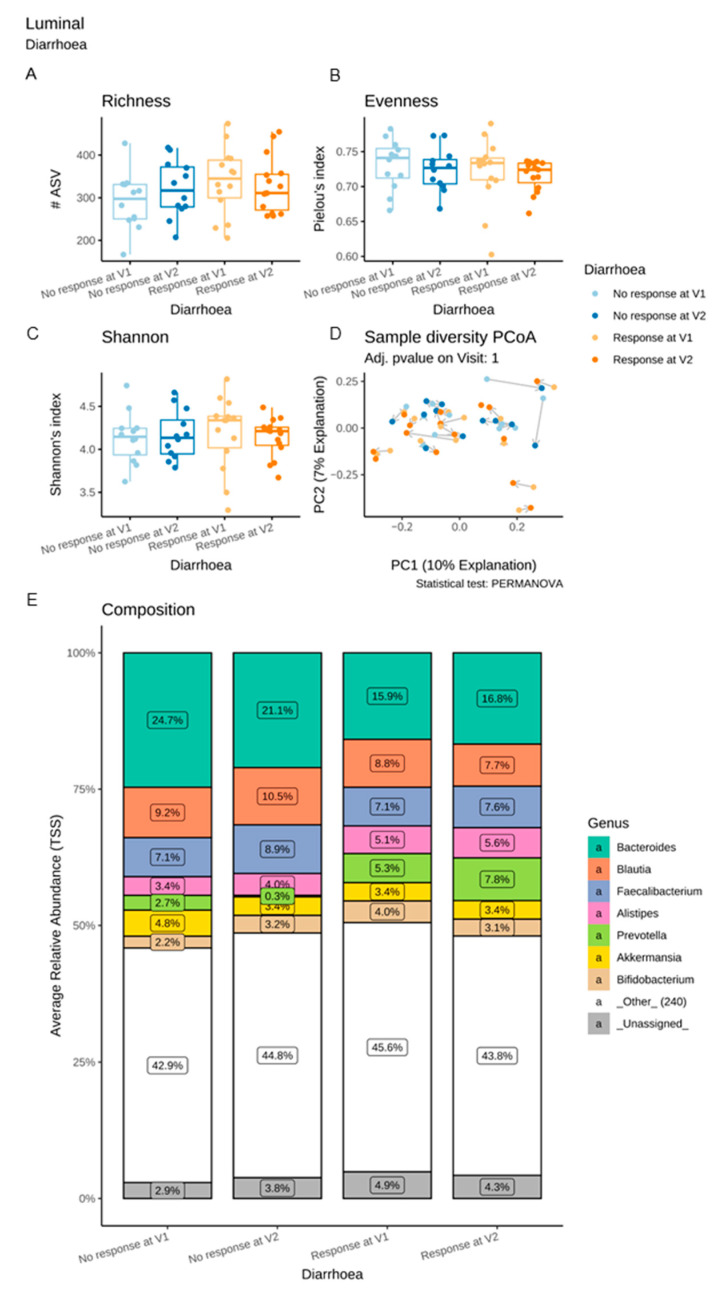
Impact of a 4-week supplementation with the multistrain probiotic on the luminal gut microbiota in Diarrhoea responders (R) and non-responders (NR). (**A**–**C**) α-diversity, estimated by the richness (number of observed ASVs) (**A**), evenness (Pielou’s index) (**B**) and Shannon’s index (**C**); (**D**) principal coordinate analysis (PCoA) of bacterial β-diversity, measured according to the Bray–Curtis distance; (**E**) bacterial taxonomic profile at genus level before (V1) and after (V2) supplementation.

**Figure 9:**
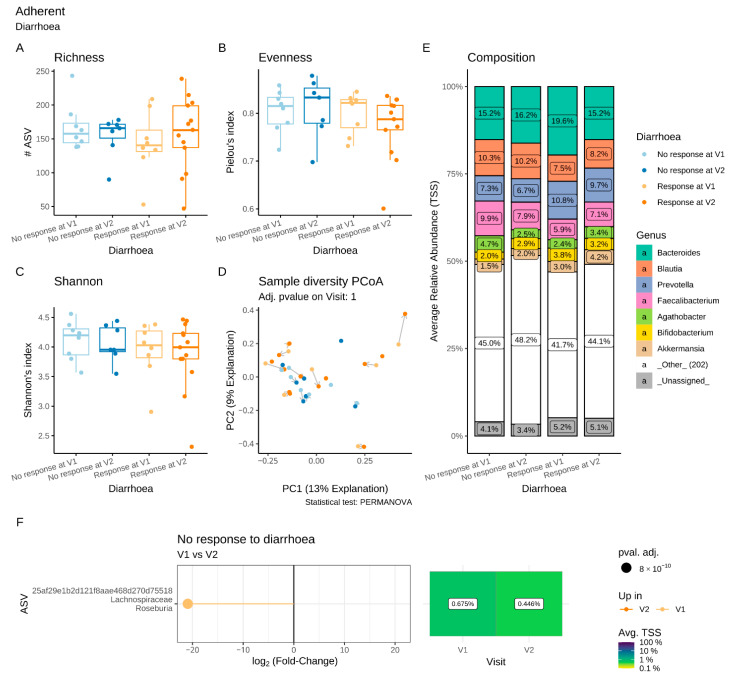
Impact of a 4-week supplementation with the multistrain probiotic on the adherent gut microbiota in Diarrhoea responders (R) and non-responders (NR). (**A**–**C**) α-diversity, estimated by the richness (number of observed ASVs) (**A**), evenness (Pielou’s index) (**B**) and Shannon’s index (**C**); (**D**) principal coordinate analysis (PCoA) of bacterial β-diversity, measured according to the Bray–Curtis distance; (**E**) bacterial taxonomic profile at the genus level before (V1) and after (V2) supplementation; (**F**) differential abundance analysis at ASV levels at V1 and V2 in Diarrhoea NR. Only significant data are presented as Log2 of the fold of change (FC) according to DESeq2 analysis. *Transcription start sites (TSS)*.

With regard to both the luminal and the adherent microbiota, the α-diversity and β-diversity indices were similar in Diarrhoea R and Diarrhoea NR at V1 and V2 (Figure 8 and Figure 9A–D). Differential abundance analyses revealed no major difference in the luminal microbiota composition in Diarrhoea R between V1 and V2 (Figure 8E). In contrast, a significant reduction of *Roseburia* was observed in the adherent microbiota of Diarrhoea NR between these two visits (Figure 9E,F).

**Figure 10:**
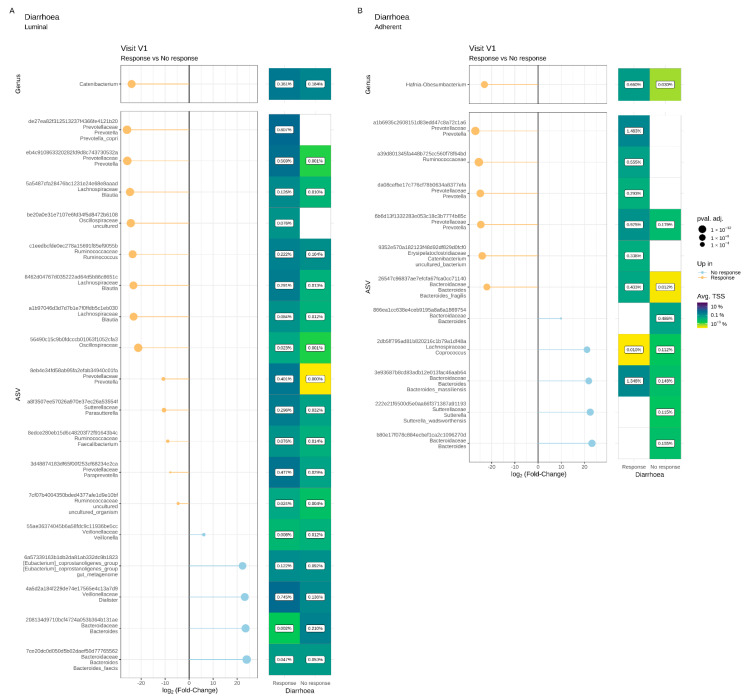
Differential abundance analysis of the luminal (**A**) and adherent (**B**) microbiota at genus and ASV levels prior to a 4-week supplementation with the multistrain probiotic in Diarrhoea responders and non-responders. Only significant data are presented as Log2 of the fold of change (FC) according to DESeq2 analysis. *Transcription start sites (TSS)*.

A luminal microbiota signature predictive of the diarrhoea response was identified at V1. As compared to Diarrhoea NR, Diarrhoea R showed a significant increase in ASVs belonging to the *Catenibacterium* genus (Figure 10A). Thirteen ASVs assigned to various families (*Prevotellacaea*, *Lachnospriraceae*, *Ruminococcaceae* and *Oscillospiraceae*) were also increased in Diarrhoea R compared to NR at V1, while five ASVs (mainly assigned to *Bacteroides* and *Veillonellaceae*) were more abundantly represented in Diarrhoea NR than in Diarrhoea R.

Similarly, the differential abundance analysis highlighted a putative adherent microbiota signature predictive of the diarrhoea response at V1 (Figure 10B). At the genus level, Diarrhoea R showed a significant increase in *Hafnia-Obesumbacteria*. Six ASVs assigned to various families, genera and species (*Ruminococcaceae*, *Catenibacterium*, *Prevotella*, *B. fragilis*) were increased in Diarrhoea R, while five ASVs (assigned to *Bacteroides*, *Coprococcus* and *Suturella*) were enriched in Diarrhoea NR.

### 3.7. Compliance and Safety

In the ITT population, the mean compliance was 100.8 ± 5.2% (range: 82.1–111.1). Twenty-nine adverse events in 15 of 30 patients were reported during the study, of which 14 were considered potentially attributable to one of the study procedures or the supplement. Seven of these fourteen adverse events involve the digestive tract. None of the adverse events was serious, and none resulted in treatment withdrawal.

## 4. Discussion

This study showed that the evaluated multistrain probiotic exerted clinical, functional and microbial effects in patients with IBS-D. After 4 weeks of supplementation, we observed: (1) a significant reduction in IBS symptoms, including abdominal pain, discomfort and diarrhoea; (2) a significant improvement in quality of life; as well as (3) a differential impact on gut permeability and (4) specific alterations of the luminal and adherent microbiota, depending on the patient’s response in terms of pain and diarrhoea. Interestingly, we also identified microbial signatures that could predict a response or a lack of response to supplementation.

The clinical results are consistent with those of previous studies evaluating the combinations of several probiotic strains, some specifically in IBS-D patients [11,31,32]. The pathophysiology of IBS is still not fully understood; however, suggested mechanisms involve visceral hypersensitivity and disturbances of the epithelial barrier function and integrity [33,34]. Gut permeability appears to be increased, more specifically in IBS-D patients than in other IBS subtypes [33,34,35]. However, only a few studies have investigated the impact of probiotics on gut permeability in IBS patients [36,37,38]. In this study, despite an improvement in clinical parameters, we did not find significant modifications in permeability in the IBS-D patient population as a whole. However, when patients were stratified according to their clinical response with regard to diarrhoea or abdominal pain, Diarrhoea R showed a reduced colonic permeability following supplementation, whereas no change was observed in Pain R. An observational study similar to ours also showed a reduction in the permeability measured with radionuclide tracers in more than 80% of IBS-D patients after supplementation with the same multistrain probiotic [39]. Probiotics may act on permeability through the modulation of tight junction expression, inflammation and mucus secretion [33]. Aside from modulation of the host physiology, probiotic beneficial effects might also reflect their ability to modulate the gut microbiota, which was reported to be altered in IBS-D [3,4,5,40]. However, most studies did not investigate either the luminal or the adherent microbiota of IBS patients during probiotic supplementation. A major novelty of our study was the complementary analysis of both the adherent and luminal gut microbiota performed before and after supplementation. We demonstrated that supplementation with the tested multistrain probiotic enriched the luminal gut microbiota of the population of IBS-D patients as a whole, with two ASVs assigned to the *Lactobacillus* and *Bifidobacterium* strains of the probiotic as previously described [36]. However, no changes in the α- and β-diversity of the luminal microbiota or the adherent microbiota diversity and composition were observed after supplementation, despite improved IBS symptoms, as previously described [36,41,42,43]. This suggests that the benefits of probiotic supplementation may be due not only to the changes in bacterial abundance but also to other mechanisms, including the action of bacteria-derived metabolites.

Interestingly, we detected specific changes in the composition of the luminal and adherent microbiota of Pain R and Diarrhoea R following supplementation. In Pain R, the multistrain probiotic induced the enrichment of the *Prevotellaceae* NK3B31 group in the luminal microbiota. A previous study in a porcine model showed that supplementation with an *L. plantarum* strain (a strain of the same species as that which was included in the multistrain probiotic evaluated in our study) induced an increased abundance of the *Prevotellaceae* NK3B31 group, which was associated with the biosynthesis of secondary metabolites, such as acetate, and intestinal maturation [44]. Most of the ASVs that were found to be increased in the luminal and adherent microbiota of Pain R belonged to the *Clostridia* class. *Clostridia* spp. has been reported to be capable of producing short-chain fatty acids (SCFAs), including acetate and butyrate, which are involved in intestinal barrier function, inflammation and the maturation of the immune system [45]. The ability of the tested multistrain probiotic to modify the gut microbiota towards a *beneficial* phenotype was only observed in Pain R. In Diarrhoea R, no significant changes in the diversity or composition of either the luminal or the adherent microbiota were detected, although major changes in permeability were observed following supplementation. In contrast, we found that the multistrain probiotic induced a decreased prevalence of an ASV belonging to *Roseburia* in the adherent microbiota of Diarrhoea NR. This suggests that lactic acid bacteria may improve IBS symptoms (pain and diarrhoea) through different mechanisms and that the initial microbiota composition may influence the response to probiotic supplementation [43].

The analysis of the microbiota prior to supplementation revealed microbial signatures that could be predictive of a response or non-response to the tested multistrain probiotic and be specific to the type of response, i.e., abdominal pain versus diarrhoea. We identified a major signature of the non-response in terms of pain in both the adherent and the luminal microbiota of IBS-D patients. Higher levels of bacteria belonging to the *Actinobacteriota* phylum (*Coriobacteriaceae UCG-002* and *Senegalimassilia*), as well as the *Clostridia* (the genera *Subdoligranulum* and *Peptococcus*) and the *Negativicutes* class (*Acidaminococcus* genus), were found in the luminal microbiota of Pain NR. Additionally, ASVs belonging to the *Bacteroidota* phylum (*Prevotellaceae* and *Rikenellaceae* families) were also more abundant in the luminal microbiota of Pain NR. A similar enrichment was observed in the adherent microbiota of Pain NR. A microbial signature with a higher level of *Actinobacteria* and lower *Alistipes* has also been identified in non-responders to the faecal microbiota transplantation (FMT) at baseline [46,47]. The higher prevalence of bacteria belonging to the same phyla as probiotic strains (*Firmicutes* and *Actinobacteriota*) or strains associated with the production of SCFA [45] suggests that Pain NR may have had a less altered microbiota than Pain R before supplementation.

Conversely, the microbial signature identified in the Diarrhoea subgroup indicated several bacteria that could predict a patient’s response to supplementation. At V1, both the luminal and the adherent microbiota of Diarrhoea R were dominated by the ASVs belonging to the *Bacteroidota* phylum (*Prevotellaceae* and *Bacteroides*). Specifically, the *Catenibacterium* genus and ASVs belonging to *Blautia* spp. were enriched in the luminal microbiota, while the *Obesumbacterium* genus was found in the adherent microbiota of Diarrhoea R. These observations are consistent with the higher prevalence of *Bacteroides* and *Blautia* reported in IBS patients and are associated, respectively, with low-grade inflammation and bowel symptoms (diarrhoea) [40,48]. The harmful properties of these genera are probably due to enterotoxigenic strains such as *B. fragilis* and their ability to produce toxins or bacteriocins [40,48]. *Obesumbacterium* has been described as a common pathogen, although the pathogenicity of these bacteria remains unclear [49]. Interestingly, a higher prevalence of bacteria belonging to the *Catenibacterium* genus and *B. fragilis* was identified in IBS-D responders with regard to both pain and diarrhoea. *B. fragilis* abundance was also found to be increased in the baseline microbiota of responders to FMT in IBS patients [47] and may represent a biomarker to predict a patient’s response to microbial therapy. Further analyses will be required to better characterise the microbial signatures or biomarkers associated with the different IBS symptoms.

Our study presents several limitations. First, it was an open-label, single-centre study, which implies a limited number of included patients, a lack of randomization, and the absence of a placebo or other control group. The interpretation of its results should therefore consider these limitations. Further evaluation of the multistrain probiotic in larger patient populations, including a control group, as well as a longer follow-up period, will therefore be important.

## 5. Conclusions

This study provides several insights into how the tested multistrain probiotic, Lactibiane Tolérance^®^, may improve IBS symptoms. In addition, analyses of both the adherent and the luminal microbiota of patients stratified by their clinical response not only revealed the distinct effects of the multistrain probiotic on the microbiota according to this response but also showed that the gut microbiota composition at baseline could predict the response or non-response to supplementation. This highlights the need to develop individualised probiotic-based approaches in patients suffering from IBS.

## Figures and Tables

**Table 1:** Impact of a 4-week supplementation with the multistrain probiotic on quality of life, assessed by the Gastrointestinal Quality of Life Index (GIQLI). Global GIQLI score and scores obtained for its sub-dimensions (mean ± SD).

	V1	V2	*p*
Global	60.6 ± 14.4	68.7 ± 14.95	<0.0001
Sub-dimensions			
Symptom	59.6 ± 12.3	68.6 ± 14.5	<0.0001
Emotion	61.9 ± 18.0	72.2 ± 20.8	0.0008
Physical function	54.1 ± 23.4	60.7 ± 22.0	0.007
Social function	67.8 ± 23.3	73.0 ± 21.6	0.03
Medical treatment	98.8 ± 5.5	93.0 ± 17.0	0.16

## Data Availability

Data can be provided upon request to the corresponding author.

## Supplementary Materials

The following supporting information can be downloaded at: https://www.mdpi.com/article/10.3390/microorganisms11020277/s1, Table S1: Mucosal response to a 4-week supplementation with the multistrain probiotic in the per protocol population: crypt architecture, density, distribution and vessels in confocal endomicroscopy. Data are expressed as mean ± SD.
