# Supplementary material for: Could the Microbiota Be a Predictive Factor for the Clinical Response to Probiotic Supplementation in IBS-D? A Cohort Study
Source: Microorganisms. 2023 Jan 20;11(2):277. doi: 10.3390/microorganisms11020277 (PMC9964083; doi:10.3390/microorganisms11020277)
Supplement: Supplementary file 1 [file microorganisms-11-00277-s001.zip › microorganisms-2065077-supplementary.pdf]

Marchix et al_Microorganisms

Supplementary material

Table S1: Mucosal response to a 4-week supplementation with the multistrain probiotic in the per protocol population: crypt architecture, density, distribution and vessels in confocal endomicroscopy. Data are expressed as mean ± SD.

	Global population	
	**V1**	**V2**
**Perimeter (px)**	1510±590	1504±477
**Sphericity (%)**	41.5±8.9	43±10
**Roundness (%)**	48.1±13.0	50.4±10.5
**Maximal Feret diameter (px)**	243.4±39.6	243.3±33.6
**Elongation factor (ratio)**	1.4±0.1	1.4±0.1
**Ma/ma ratio**	1.2±0.1	1.2±0.1
**Crypt density (ratio)**	7.45±1.96	7.93±1.60
**Minimal ICD (px)**	548±116	557±147
**Mean ICD (px)**	335±55	351±58
**Wall thickness (µm)**	365±122	369±140
**Mean vessel area (%)**	0.17±0.04	0.17±0.04
**Mean vessel diameter (µm)**	12.5±0.74	12.45±0.60
